# Detection of SARS-CoV-2 RNA in Urine by RT-LAMP: A Very Rare Finding

**DOI:** 10.3390/jcm11010158

**Published:** 2021-12-29

**Authors:** Juan García-Bernalt Diego, Pedro Fernández-Soto, Juan Luis Muñoz-Bellido, Begoña Febrer-Sendra, Beatriz Crego-Vicente, Cristina Carbonell, Amparo López-Bernús, Miguel Marcos, Moncef Belhassen-García, Antonio Muro

**Affiliations:** 1 Infectious and Tropical Diseases Research Group (e-INTRO), Research Centre for Tropical Diseases at the University of Salamanca (IBSAL-CIETUS), Biomedical Research Institute of Salamanca, Faculty of Pharmacy, University of Salamanca, 37007 Salamanca, Spain; juanbernalt95@usal.es (J.G.-B.D.); begofebrer@usal.es (B.F.-S.); beatrizcregovic@usal.es (B.C.-V.); carbonell@usal.es (C.C.); alopezb@saludcastillayleon.es (A.L.-B.); belhassen@usal.es (M.B.-G.); 2 Microbiology and Parasitology Service, Complejo Asistencial Universitario de Salamanca (CAUSA), University of Salamanca, 37007 Salamanca, Spain; jlmubel@usal.es; 3 Internal Medicine Service, Unidad de Enfermedades Infecciosas, Complejo Asistencial Universitario de Salamanca (CAUSA), University of Salamanca, 37007 Salamanca, Spain; mmarcos@usal.es

**Keywords:** SARS-CoV-2, urine, COVID-19, RT-LAMP, RT-qPCR, molecular diagnostics

## Abstract

Detection of SARS-CoV-2 is routinely performed in naso/oropharyngeal swabs samples from patients via RT-qPCR. The RT-LAMP technology has also been used for viral RNA detection in respiratory specimens with both high sensitivity and specificity. Recently, we developed a novel RT-LAMP test for SARS-CoV-2 RNA detection in nasopharyngeal swab specimens (named, N15-RT-LAMP) that can be performed as a single-tube colorimetric method, in a real-time platform, and as dry-LAMP. To date, there has been very little success in detecting SARS-CoV-2 RNA in urine by RT-qPCR, and the information regarding urine viral excretion is still scarce and not comprehensive. Here, we tested our N15-RT-LAMP on the urine of 300 patients admitted to the Hospital of Salamanca, Spain with clinical suspicion of COVID-19, who had a nasopharyngeal swab RT-qPCR-positive (*n* = 100), negative (*n* = 100), and positive with disease recovery (*n* = 100) result. The positive group was also tested by RT-qPCR for comparison to N15-RT-LAMP. Only a 4% positivity rate was found in the positive group via colorimetric N15-RT-LAMP and 2% via RT-qPCR. Our results are consistent with those obtained in other studies that the presence of SARS-CoV-2 RNA in urine is a very rare finding. The absence of SARS-CoV-2 RNA in urine in the recovered patients might suggest that the urinary route is very rarely used for viral particle clearance.

## 1. Introduction

The infection caused by SARS-CoV-2 (COVID-19) affects mainly the respiratory system, and the typical symptoms at onset are fever, dry cough, fatigue, and dyspnea. Most patients present a good prognosis, while a few develop severe complications such as acute respiratory distress syndrome (ARDS) which can lead to death [1,2]. SARS-CoV-2 infects human cells using the angiotensin-converting enzyme 2 (ACE2) receptor [3,4,5]. Due to the expression across a wide variety of human tissues of ACE2, COVID-19 infection can lead to renal, hematological, skin, neurological, cardiovascular, gastrointestinal, liver, endocrine, and ophthalmological involvement, with different degrees of severity in patients [6,7]. The potential systemic dissemination of SARS-CoV-2 suggests that detection of viral particles or viral RNA might be possible in several biologic fluids depending on the patients’ disease presentation.

Nucleic acid amplification tests (NAATs) are considered the most appropriate methods for screening suspected SARS-CoV-2 cases, with the reverse transcription-polymerase chain reaction (RT-qPCR) the gold standard test to confirm infection [8,9]. Respiratory samples are considered the most efficient specimens for the isolation and detection of SARS-CoV-2 RNA, particularly upper respiratory tract samples (nasopharyngeal or oropharyngeal swabs) in the early stages of infection, and lower respiratory tract samples in the later stages of infection (mainly sputum, if produced) [8]. Other clinical specimens such us saliva, stool, urine, serum, blood, tears [10,11,12] and seminal fluid [13] have been tested for the presence of SARS-CoV-2 with varying degrees of efficiency.

Regarding urine, despite being an easy sample to obtain, less intrusive and with less potential risk of infection for health workers, to date, there has been very little success in detecting viral RNA by RT-qPCR [11]. In fact, the evidence regarding the presence, viability, and infectivity of SARS-CoV-2 in urine is sparse. Additionally, the few methodologically sound studies using RT-qPCR for detection included a small number of specimens and found a very low rate of positivity [14]. On the other hand, there are still conflicting opinions as to whether the presence of the virus in urine can be an indicator of severity or worse prognosis of the disease [15,16,17,18,19].

A recent study has shown that the spike protein of SARS-CoV-2, particularly subunit S1, can be detected in the urine of patients with a nasopharyngeal RT-qPCR-positive result using an antigen-capture assay. However, sensitivity of this methodology is highly reduced compared to nasopharyngeal RT-qPCR (only 25% of SARS-CoV-2 infected individuals are detected) [20].

In addition, the RT-qPCR methodology is not easily adaptable for point-of-care diagnosis in situations requiring rapid diagnosis. To solve this, an increasingly recognized alternative is loop-mediated isothermal amplification (LAMP) technology. Since the LAMP technique was first described [21], it has been used to detect a target nucleic acid (DNA or RNA) mainly for molecular diagnostics purposes without sophisticated equipment. LAMP can amplify DNA from an RNA sample (RT-LAMP) in a one-step reaction by the addition of a reverse transcriptase to the LAMP reaction or a DNA-polymerase with retrotranscriptase activity [22]. In addition, the LAMP assay can operate at different pH and temperature ranges and with crude samples, which is advantageous for working with multiple clinical samples [23]. In this regard, it has been used for the detection of SARS-CoV-2 RNA, with sensitivities well over 90% when using purified RNA samples [24] and reaching 85% when using unprocessed pharyngeal samples [25]. Furthermore, a color RT-LAMP based diagnostic assay has been recently approved for emergency use by the U.S. Food and Drug Administration (FDA) for COVID-19 diagnostics [26].

Recently, we developed an RT-LAMP test for SARS-CoV-2 RNA detection in clinical nasopharyngeal swabs specimens by targeting gene N with a specific-sequence primer set N15 (N15-RT-LAMP) that can be performed as a single-tube colorimetric method, in a real-time platform, and as dry-LAMP. This assay proved to be specific for SARS-CoV-2 and showed a limit of detection of 200 copies per reaction (cpr) [27]. The RT-LAMP methodology has already been tested successfully in the detection of SARS-CoV-2 RNA in urine but only using artificially spiked samples with various concentrations of SARS-CoV-2 RNA [28]. However, to the best of our knowledge, there are no clinical studies evaluating the usefulness of RT-LAMP as a SARS-CoV-2 detection molecular tool in COVID-19 patients’ urine samples. Thus, the objective of the present study was to evaluate the usefulness of RT-LAMP for the detection of SARS-CoV-2 RNA in urine samples. To do so, we tested our N15-RT-LAMP method on urine of 300 patients (including COVID-19 positive, negative and recovered patients) admitted with COVID-19 clinical suspicion at the Hospital of Salamanca, Spain. We also compared N15-RT-LAMP results in urine samples with those obtained by RT-qPCR in nasopharyngeal swabs. Additionally, for the first time, urine samples from a recovered group of patients were evaluated by RT-LAMP for the presence of SARS-CoV-2 RNA.

## 2. Materials and Methods

### 2.1. Nasopharyngeal Specimen Collection, RNA Isolation and RT-qPCR

Nasopharyngeal swab specimens were collected from admitted patients of the University Hospital of Salamanca, Salamanca, Spain, with compatible COVID-19 symptoms. Collected samples were first preserved in sample preservation solution (MOLE BIOSCIENCE, SUNGO Europe B.V., Amsterdam, The Netherlands), delivered to the Laboratory of Microbiology, and then processed in a biosafety level 2 cabin until inactivation by mixing with a lysis buffer. Nasopharyngeal swabs were processed in an integrated platform for both RNA isolation and RT-qPCR (COBAS 6800, ROCHE, Basel, Switzerland), targeting ORF1ab and E gene of SARS-CoV-2, following the manufacturer’s instructions.

### 2.2. Urine Specimen Collection, RNA Isolation and RT-qPCR Analysis

**Figure 1:**
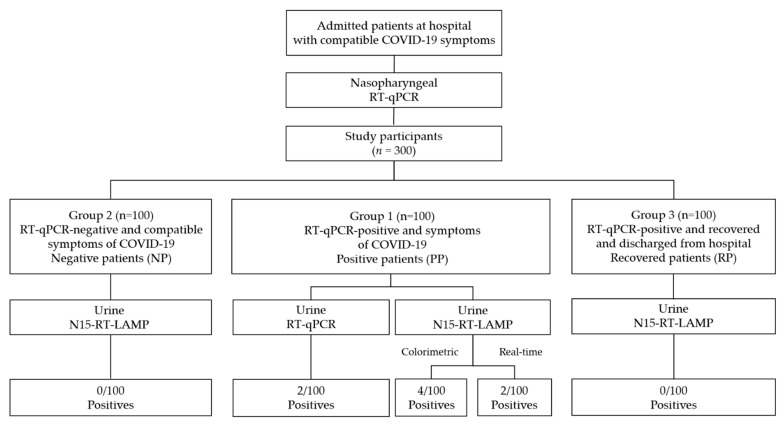
Flowchart describing the process of patient group selection and urine sample analysis strategy. RT-qPCR, reverse transcription-polymerase chain reaction; RT-LAMP, reverse transcription loop-mediated isothermal amplification.

Along with the nasopharyngeal samples, urine samples for routine analysis were also collected from inpatients. An aliquot of these samples was reserved for COVID-19 analysis. Based on the results obtained from the nasopharyngeal swabs RT-qPCR tests, a total of 300 patient urine samples were selected and grouped as follows (Figure 1): Group 1: 100 urine samples from patients with a RT-qPCR positive result and symptoms of COVID-19 (PP, positive patients); Group 2: 100 urine samples from patients with a RT-qPCR negative result, but with compatible symptoms of disease (NP, negative patients); and Group 3: 100 urine samples from symptomatic patients with RT-qPCR positive on admission, but recovered and discharged from hospital (RP, recovered patients). PP urine samples were obtained within 24 h of a positive result by RT-qPCR in nasopharyngeal swabs; RP urine samples were obtained 7 days after hospital discharge, with a negative RT-qPCR result in nasopharyngeal swabs. Patients selected in the study presented the typical symptoms described for COVID-19 including cough, fatigue, sputum production, sore throat, headache and shortness of breath, among the most frequent [1].

Urine samples were individually collected in conventional sterile urine containers and delivered to the e-INTRO group’s laboratory for further processing in a biosafety level 2 cabin until inactivation by mixing with the lysis buffer. Subsequently, 1 mL from each urine sample was concentrated to a final volume of 200 µL that was used as the input for RNA extraction using the NZY Viral RNA Isolation Kit (NZYTECH, Lisbon, Portugal) following the manufacturer’s instructions. Afterwards, the 100 urine samples from the PP group were analyzed by RT-qPCR using the SARS-CoV-2 One-Step RT-PCR Kit, CE-IVD (NZYTECH, Lisbon, Portugal) following the recommended conditions, and viral copy numbers were calculated according to the manufacturer’s instructions.

### 2.3. RT-LAMP Analysis

All 300 RNA isolates from urine samples were analyzed by a one-step conventional colorimetric RT-LAMP targeting a region of 212 base pairs (bp) of the N gene of SARS-CoV-2, recently described by our group and referred as N15-RT-LAMP [27]. In brief, colorimetric N15-RT-LAMP assays were performed in the presence of two enzymes: *Bst* 2.0 WarmStart DNA polymerase (*Bst* 2.0 WS) and WarmStart RTx reverse transcriptase (RTx WS) (NEW ENGLAND BIOLABS Ltd., Ipswich, MA, USA) in a volume of 15 μL containing 1.6 μM FIP/BIP primers, 0.2 μM F3/B3 primers, 0.4 μM LF/LB primers, 1.4 mM of each dNTP (BIORON GmBH, Römerberg, Germany), 0.13 M of D-(+)-trehalose dihydrate (Sigma-Aldrich, St. Louis, MO, USA) (hereinafter trehalose, for short), 6 mM MgSO_4_, 1× amplification buffer (20 mM Tris-HCl (pH 8.8), 50 mM KCl, 10 mM (NH_4_)_2_SO_4_, 2 mM MgSO_4_, 0.1% Tween20), *Bst* 2.0 WS (0.32 U/μL) and *RTx* WS (0.3 μL), with 2 μL of template RNA. All RT-LAMP reactions were performed at 63 °C for 45 min in a heating block, followed by 10 min at 80 °C for enzyme inactivation. Results were evaluated by the naked eye using 1 μL of SYBR Green I 1000× (INVITROGEN, Carlsbad, CA, USA) added post-amplification to each reaction tube. For positive results, the dye turns to an intense green/yellow, whilst negative reactions remain orange. To avoid potential post-amplification contamination, each tube was briefly centrifuged and carefully opened in a laminar flow hood to add the intercalating dye.

In addition, real-time N15-RT-LAMP assays were performed in 8-tube Genie Strips on a portable Genie III device (OPTIGENE Ltd., Horsham, UK) using the same reaction mixes as previously described but adding 0.24 μL of EvaGreen 20× in water (BIOTIUM, San Francisco, CA, USA) before the reaction started. In all N15-RT-LAMP assays, positive (2 μL of RNA purified from a nasopharyngeal swab from a patient with a RT-qPCR-positive result for SARS-CoV-2; Ct = 25 for ORF1ab) and negative (2 μL of ultrapure water) controls were included. All positive results were confirmed in triplicates.

## 3. Results

As stated above, the 300 patients included in this study were divided into three groups of 100 individuals each according to the nasopharyngeal swabs RT-qPCR testing: PP, NP, and RP. The gender ratio in the study was 51.67% female and 48.33% male, with a higher proportion of women in NP group (57% vs. 43%) than in PP group (39% vs. 61%). Overall, the mean age was 66.47 years (standard deviation (SD) = ±16.78), with a minimum age of 20 and a maximum of 99, which was lower in RP group (64.90 ± 13.60) than in PP group (68.14 ± 17.80).

**Figure 2:**
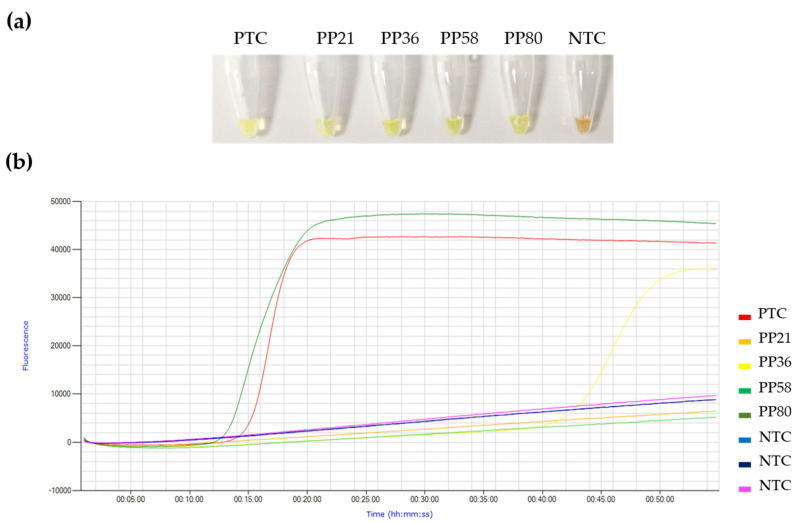
Detection of SARS-CoV-2 RNA in urine samples by colorimetric and real-time N15-RT-LAMP. (**a**) Colorimetric N15-RT-LAMP: results detected with SYBR Green I. Green/yellow, positive results; orange, negative results. (**b**) Real-time N15-RT-LAMP: results detected by fluorescence in a Genie III device. PTC, positive template control (2 μL of RNA purified from a nasopharyngeal swab from a patient with a RT-qPCR-positive result for SARS-CoV-2; Ct = 25 for ORF1ab); PP21, PP36, PP58, and PP80, patient urine samples; NTC, negative template control (2 μL of ultrapure water instead RNA).

First, the 100 urine RNA isolates from the PP group were analyzed by RT-qPCR in our laboratory (see Figure 1). Only 2/100 (2%) were found positive (nos. PP80 and PP36), with cycle threshold (Ct) values resulting in 23.65 and 36.76, respectively. Then, all the 300 urine RNA isolates were tested by our conventional colorimetric N15-RT-LAMP and 4/100 (4%) samples were positive in PP group (nos. PP21, PP36, PP58, and PP80), including the 2 positive samples previously obtained by RT-qPCR analysis (see Figure 1 and Figure 2a). No positives were detected in the NP group (0/100) or in the RP group (0/100).

The 4 urine RNA positive samples were also tested by real-time N15-RT-LAMP for a better comparison with RT-qPCR results (Figure 2b). Only the 2 RT-qPCR positives (nos. PP80 and PP36) were also positive by real-time N15-RT-LAMP, with a time to positivity (Tp) values of 14.5 min and 45 min, corresponding to Ct values by RT-qPCR of 23.65 and 36.76, respectively.

Relevant available clinical data from the four positive patients are presented in Table 1. After an analysis of the symptoms, clinical presentation, and medical history of the four positive participants, no relevant common clinical pattern was found.

## 4. Discussion

At present, information on SARS-CoV-2 is already extensive, but urine viral excretion tests regarding both the presence and virulence of the virus are still very scarce. Although the WHO has recommended in its guidelines to consider urine testing for all symptomatic patients and contact persons [8], the presence of SARS-CoV-2 in urine samples is still poorly investigated in the current literature and a few methodologically sound studies have reported only a very low rate of positivity [14]. It should be noted that, despite the rarity of the event, it can occur. The results from our study reinforce this point.

Remarkably, out of a group of 100 patients admitted to hospital with COVID-19 symptoms and a nasopharyngeal swab RT-qPCR-positive result, only two (2/100; 2%) tested positive for the presence of SARS-CoV-2 RNA by RT-qPCR when urine samples were analyzed. In these two positive samples, a great variation in viral load was detected, with one sample having a viral copy number of around 500,000 copies/mL, and the other just 50 copies/mL. Notwithstanding the difference in viral load, no differences in the severity of COVID-19 were observed in these patients.

A study performed by Yu et al. [29] using droplet digital PCR (ddPCR) for an accurate quantification of viral load, showed great variation in viral load among COVID-19 patients. In that study, the viral loads in the early and progressive stages of COVID-19 were significantly higher (over 46,000 copies) than in the recovery stage of the disease (over 1200 copies) when analyzing different types of samples, including nasal swabs, throat swabs, sputum, blood and urine. However, no positive results were found in blood or urine. To date, only two studies have provided the viral copy number of SARS-CoV-2 detected by RT-qPCR in urine. In a study by Peng et al. [30], a concentration of 322 copies/mL was found in the urine of a patient with COVID-19 symptoms. In another study by Frithiof et al. [16], a mean concentration of 1200 copies/mL (range 300–2800) was found in six critically ill COVID-19 patients. The paucity of previous studies makes comparison with our results difficult. Nevertheless, the data seem to indicate that the number of viral copies in the urine of patients with COVID-19 symptoms may be highly variable, as was also the case in our two RT-qPCR-positive patients.

In the overall analysis, our conventional colorimetric N15-RT-LAMP detected four positive urine samples from PP group, including the two samples positive by RT-qPCR. Although slight, this higher positivity rate (4% vs. 2% by RT-qPCR) suggests a higher sensitivity of our LAMP method over RT-qPCR for analysis of RNA isolates from urine. Unexpectedly, this higher positivity rate was not found when N15-RT-LAMP was performed under real-time conditions, as identical results were obtained on the same two RT-qPCR-positive samples. The fact that two urine samples were positive by conventional colorimetric N15-RT-LAMP, but not in real-time settings, could be due to the presence of pre-amplified EvaGreen fluorescent dye in the reaction mixes for real-time monitoring. It has been shown in LAMP assays that EvaGreen can produce a partial inhibition of the reaction by reducing both the reaction rate and final amplification levels [31]. This does not occur in conventional colorimetric LAMP assay since the SYBR Green I dye is added when the amplification is already finished. This would be an advantage for analysis since only a heating block would be needed to carry out the reaction. Another possible explanation for the amplification of these two samples by conventional, but not real-time, N15-RT-LAMP, could have been non-specific amplification or post-amplification contamination by opening the tubes and adding SYBR Green I at the end of the reaction. However, we sincerely believe that the specificity demonstrated by our N15-RT-LAMP in its previous development and set up [27], the gentle handling of the tubes in a laminar flow hood, and the triplicate confirmation of positive results, rule out those possibilities.

The current gold standard for diagnosing COVID-19 is based on molecular tests of RT-qPCR to detect the RNA of the virus in respiratory samples such as nasopharyngeal swabs or bronchial aspirate using different protocols and target sequences available in the WHO database [8,32]. However, it is important to note that RT-qPCR is not fail-safe and can also give false negatives if viral load is low or if the correct time-window of viral replication is missed. In this sense, the COVID-19 incubation period is estimated to be 5 days, but false negative results may occur within the first week of infection [33]. In addition, possible sources of RT-qPCR false results can include laboratory errors at three different stages including, pre-analytical, analytical and post-analytical phases [34]. Furthermore, the commercially available diagnostics kits in RT-qPCR have different features, mainly connected to the viral target tested and the limit of detection. Significantly, the higher the limit of detection, the more risk of false negatives [35].

A recent systematic review with meta-analysis conducted by Böger et al. [36]. compared the clinical performance of RT-qPCR tests for SARS-CoV-2 detection using different samples (including oral saliva, deep-throat saliva, posterior oropharyngeal saliva, sputum, urine, feces, and tears) against standard specimens (nasopharyngeal and oropharyngeal swabs). As a result, oral saliva proved to be the most promising sample for the detection of SARS-CoV-2, not only because of the high diagnostic accuracy obtained (above 90%), but also because it allows self-collection (decreasing the risk of exposure of health-care workers to infections) and reduces the waiting time for sample collection (favoring epidemiological measures). Unfortunately, for urine, the authors did not find enough studies to calculate estimates and, yet again, no data are provided in meta-analysis making comparison with our results impossible.

To date, the only systematic review of the literature to investigate the presence of SARS-CoV-2 specifically in human urine is the one conducted by Bröniman et al. [14]. The study concluded that: (i) this finding is still poorly investigated (0.6%; 34/5674 articles); (ii) only 7 studies reported positive results with a very low rate of positivity and, (iii) 90% of the patients with multiple urine analysis displayed a positive RT-qPCR only at one single point in time. On the other hand, some studies have indicated that, in addition to being a rare occurrence, urinary viral secretion was not associated with acute kidney injury or severity of COVID-19 disease [15,16]. However, several other studies have found that those subjects with SARS-CoV-2 in the urine at admission to hospital and its persistence during hospitalization had more severe COVID-19 [18,19] and a worse clinical course [19]. Thus, there are different studies with conflicting results regarding the presence of SARS-CoV-2 RNA in urine and the progression of COVID-19. However, all these studies are based on very limited sample sizes and agree in recognizing the need for more large-scale studies to better assess this hypothesis and possible future implications. In our study, we found a low positivity rate in urine samples (4%) in the PP group. Among the four positive patients, the one with the highest viral load (PP80; see Table 1) had the worst prognosis (death). Nonetheless, stage III renal insufficiency and the advanced age of the patient could explain both the presence of RNA in urine and the disease prognosis. Apart from these observations, no common clinical features were found among all four positives.

We acknowledge the limitations of our study. The study lacks viral load data from patients’ nasopharyngeal samples, which makes it difficult to contextualize urine-positive samples within the positive group of patients. In addition, plasma samples could have been informative for these positive results. Although several studies have included plasma for the detection of SARS-CoV-2 by nucleic acid testing with variable results (mainly in the early stages of the disease, raising doubts about its diagnostic value) it could be of added value in determining or predicting the severity of COVID-19 [37]. Finally, due to the very limited positive results, our study lacks strong statistical analysis.

Overall, compared to previous studies on the molecular detection of SARS-CoV-2 RNA in human urine, our work using N15-RT-LAMP technology provides a larger number of urine samples analyzed from three groups of patients with COVID-19, including symptomatic positives, symptom-compatible negatives and, for the first time, patients recovered from the disease. Our results are consistent with those obtained in other studies in finding that the presence of SARS-CoV-2 RNA in urine is highly unlikely. Additionally, the absence of SARS-CoV-2 RNA in urine in our recovered patients could indicate that the urinary route is very rarely used for the clearance of viral particles.

## Figures and Tables

**Table 1:** Most relevant clinical data of the four patients who tested positive for SARS-CoV-2 in a urine sample by N15-RT-LAMP.

	PP21	PP36	PP58	PP80
Demografic data				
Age (years)	63	73	33	92
Sex	Male	Female	Male	Male
Immunosuppression	No	Yes	No	Yes
Immunosupression cause	N/A	Diabetes mellitus	N/A	Renal insufficiency
Previous clinical data				
Non-COVID-19 Disease	Dyslipemia, Renal insufficiency I, Anxiety	Diabetes mellitus, Arterial hypertension, Cognitive impairment, Dyslipemia	Asthma, Dyslipemia	Renal insufficiency III, Ischemic heart disease, Depression
Previous medical treatment	Atorvastatine, Diazepam	Losartan, Rosuvastatine, Amantadine	Pravastatine/fenofibrate, Salbutanol	Acetylsalicylic acid, Bisopropol, Furosemide, Omeprazole
COVID-19 clinical data				
Fever	Yes	Yes	Yes	No
Dyspnea	Yes	Yes	Yes	Yes
Cough	Yes	Yes	Yes	No
Ageusia/Anosmia	No	Yes	No	No
Myalgia	Yes	No	No	No
Asthenia	No	Yes	No	No
Diarrhea	No	No	No	No
Microbiological diagnosis	Yes	Yes	Yes	Yes
RT-qPCR	Positive	Positive	Positive	Positive
Days since symptom onset	14	3	10	1
Diagnosis	Bilateral pneumonia	Bilateral pneumonia	Bilateral pneumonia	Bilateral pneumonia
Specific treatment for COVID-19				
Steroids	Yes	Yes	No	Yes
Tocilizumab	Yes	Yes	No	No
Remdesevir	No	No	No	No
Heparin	Yes	Yes	No	No
Others		Hydroxychloroquine	Amoxicillin	Lopinavir/Ritonavir-Hydroxychloroquine-Cefthriaxone
Evolution of COVID-19				
Stay (days)	10	5	0	3
ICU	No	No	No	No
Dead	No	No	No	Yes
Other diagnosis during COVID-19 stay	Lupus discoid	No	No	No
